# Self-Care and Mental Health Among College Students During the COVID-19 Pandemic: Social and Physical Environment Features of Interactions Which Impact Meaningfulness and Mitigate Loneliness

**DOI:** 10.3389/fpsyg.2022.879408

**Published:** 2022-06-16

**Authors:** Ruth Barankevich, Janet Loebach

**Affiliations:** ^1^School of Social Ecology, University of California, Irvine, Irvine, CA, United States; ^2^Department of Human Centered Design, Cornell University, Ithaca, NY, United States

**Keywords:** social interaction, self-care, loneliness, mental health, college students, physical environment, virtual interactions, COVID-19 pandemic

## Abstract

The COVID-19 pandemic has interrupted patterns and limited opportunities for social interaction, which increased already high loneliness rates among college students. Meaningful social interactions can mitigate negative mental health outcomes such as loneliness and bolster social support, which is in turn linked to better self-care practices. Social connection can aid in self-care through social support as well as be considered a self-care practice itself to counter the negative effects of loneliness. This study examined the social interaction patterns of 132 college students from a mid-sized United States university during the pandemic to understand which characteristics support meaningful interactions. Students completed an online survey from October through December 2020 to report details of their 2020 and 2019 social interactions, as well as their most recent interactions, including time spent, the mode (in-person versus virtual), their relationship to others in the interaction, the type of activity and privacy of the setting. Results found that students spent significantly less time interacting with non-roommates in-person in 2020, and more time in voice and video calls. No differences were found for texting and in-person roommate interactions. Meaningfulness was significantly higher for interactions with family or friends. Students reported the highest meaning for interactions that were planned and in-person, with lowest meaning for planned virtual interactions. No differences were observed for meaningfulness based on the type of interaction activity or privacy of the setting. Understanding the characteristics of the most meaningful interactions can help college students prioritize social interactions that may best promote self-care, mitigate loneliness, and bolster social support. High meaningfulness scores for planned in-person interactions suggests that these types of interactions may be most valuable for maintaining existing self-care patterns, engaging in self-care activities, and receiving support. Self-care activities for college students, including social interactions, were significantly impacted by the COVID-19 pandemic, which may have further exacerbated loneliness. College students should be encouraged to consciously engage in person with family and friends to practice self-care and maintain or improve mental health. Strategically selecting interactions that will optimize meaningfulness may therefore be critical to helping students to maintain positive mental health during and beyond the pandemic.

## Introduction

Scholars have been examining a rise in reported loneliness, especially among young adults, even before the beginning of the COVID-19 pandemic (Twenge et al., 2021). Social distancing restrictions and stay-at-home orders that accompanied the pandemic have only exacerbated feelings of loneliness for many (Groarke et al., 2020; Killgore et al., 2020). One of the reasons for the increase in loneliness is likely the decrease in social interactions, especially those occurring in-person, as pandemic-induced shifts in community expectations and social behaviors have been accompanied by a significant transfer to online learning, working, and socializing.

Loneliness, a feeling of inadequate social interaction or fulfilling relationships (Russell et al., 1980; Masi et al., 2011), has received growing attention in the last few decades from scholars, journalists, and governments, with media reports of loneliness becoming ubiquitous. Research has linked loneliness to a myriad of negative physical and mental health conditions, including cardiovascular disease (Hawkley and Cacioppo, 2010; Valtorta et al., 2016; Xia and Li, 2018), poor sleep quality (Cacioppo et al., 2002; Segrin and Passalacqua, 2010), depression (Cacioppo et al., 2006; Killgore et al., 2020), lower immune functioning (Kiecolt-Glaser et al., 2010; Xia and Li, 2018), and premature mortality (Hawkley and Cacioppo, 2010; Shiovitz-Ezra and Ayalon, 2010; Holt-Lunstad et al., 2015; Christensen et al., 2020). In addition to being tied to numerous negative health outcomes, loneliness feeds a lack of interest and ability to participant in positive health behaviors (Segrin and Passalacqua, 2010), including maintaining important self-care practices in daily routines that positively influence health and wellbeing (Narasimhan et al., 2019). Due to its embeddedness in a complex web of sociocultural and environmental factors, as well as individual characteristics, it can be difficult to fully understand underlying factors that contribute to loneliness (Masi et al., 2011).

Untangling the variables linked to loneliness can be additionally challenging when looking at individuals under the age of 30, as during the shift from adolescence to adulthood that are simultaneously navigating a complex world of shifting societal expectations, identity, responsibilities, social ties, and often residence (Shaver et al., 1985; Arnett et al., 2014). The transition between life phases, often highlighted by the move from high school into college, can leave young adults especially vulnerable to experiencing loneliness, with young adults between the ages of 18 and 22 reporting some of the highest rates of loneliness (Renken, 2020). These feelings can interplay with other mental health concerns, such as anxiety, and could interfere with the formation of social bonds and the development of healthy practices important for long-term happiness and health (Moore and Schultz, 1983; Mahon et al., 1998). Even though these changes in networks, activities, and physical and social spaces which young adults inhabit contribute to increased difficulty in pinpointing factors associated with loneliness, it is nevertheless vital for research to untangle and identify factors which could be targeted for interventions to help mitigate this experience.

Among several types of interventions identified in a meta-analysis of studies aimed at reducing loneliness, Masi et al. (2011) found that improving social support and increasing social interaction opportunities could be viable mechanisms for achieving this outcome. Considering the critical role of social interaction in building and enhancing social support, understanding patterns and characteristics of social interactions could help individuals create strategies for improving both social support and reducing loneliness. Social interaction is theorized to have a bi-directional relationship with loneliness, such that it is influenced by and in turn shapes social interactions (Nowland et al., 2018).

One pathway for influencing social interaction to mitigate loneliness is through promoting self-care, broadly defined as the engagement in activities that foster health and well-being, including sleep, medication adherence, and meditation (Richard and Shea, 2011; Myers et al., 2012). Self-care practices are directly and indirectly related to both social interaction and social support. Although research in this field focuses primarily to self-care in post-operative contexts, studies have found that a greater amount of social support has been linked to better self-care practices (Gallant, 2003; Vassilev et al., 2011; Chen et al., 2017). For example, individuals undergoing pulmonary rehabilitation engaged in better self-care behaviors if they had higher amounts of social support, specifically positive social interactions (Chen et al., 2017). Social interaction can influence self-care practice through encouragement from one’s social support network to employ in better self-care practices and strategies (e.g., Chen et al., 2017). Social interaction may also influence self-care more directly, when examined as a type of self-care practice, although evidence in this research area is limited. More specifically, social interaction is an activity for maintaining both physical health and emotional well-being (e.g., hampering loneliness), especially when social interactions envelop activities that directly promote health outcomes (e.g., exercise; healthy eating).

Promoting or providing the conditions conducive to social interactions may therefore be a viable mechanism to counteract the negative effects of loneliness. Although both the quantity and quality of social interactions are likely to have positive impacts on health and well-being (Fiorillo and Sabatini, 2011; Lodder et al., 2015; Luhmann and Hawkley, 2016; Sun et al., 2020), higher quality interactions are more effective in promoting positive outcomes (Luhmann and Hawkley, 2016). While an examination of the quality of interactions can be operationalized in different ways, the current study assesses quality through meaningfulness, or how meaningful an interaction is to an individual, as this variable has been found to predict loneliness (Wheeler et al., 1983). Meaningful social interactions, therefore, may impact loneliness by directly reducing lonely feelings, by helping build social support networks that can mitigate loneliness (Masi et al., 2011), and by providing self-care strategies that could counteract the negative mental and physical effects of loneliness.

The pandemic has limited social interaction opportunities and interrupted daily interaction patterns for millions of individuals around the globe. College students have been disproportionally impacted, as many students were asked to leave their campus housing and move back to their family homes, often in a different city. During most of the year 2020, places for interacting in-person with peers, mentors, and faculty members, especially on-campus and indoor public spaces, were not accessible to most college students and many classes were held virtually. This interruption undoubtedly impacted existing coping and self-care strategies that college students utilized, leading to increases in loneliness during the pandemic (Killgore et al., 2020). As the pandemic shifts to an endemic, and many restrictions related to social interactions remain, it is important that we examine the types and conditions of social interactions which were meaningful for college students during the pandemic to understand how we can support the interactions which can promote self-caring, improve social support, and mitigate loneliness during and beyond the COVID-19 pandemic.

In examining college students’ social interactions, we hypothesized that higher meaningfulness would be associated with interactions that (1) included family and friends; (2) occurred in-person as opposed to virtually, and (3) were planned in advance as compared to interactions that are spontaneous. We also anticipated that meaningfulness would be higher when interaction activities were leisurely in nature when compared to work- or study-related interactions, or a mix of both activity types. Meaningfulness was also expected to be significantly different based on the characteristics of the environmental setting in which it occurred, including the type of space and the privacy level provided by the setting.

## Materials and Methods

Undergraduate and graduate students at a mid-sized university in the northeastern United States were recruited to participate in a study examining social interactions patterns among college students during the COVID-19 pandemic. A questionnaire was administered online from October 2020 through December 2020; eligible participants were able to complete the survey at any time during the study period. While many of the students had returned to the university’s community for the Fall 2020 semester, the majority of classes were held virtually. If students spent time on campus, they were required to adhere to strict pandemic guidelines, including 6-foot distancing, limiting room occupancy, and wearing masks.

The questionnaire took approximately 25 min to complete. Participants were asked to report answer questions related to their demographics, personality type (Big 5 personality traits; Gosling et al., 2003), and location at which they were currently residing. Participants also reported details related to their interactions from the past week, including specific features of their most recent interaction. They were also asked to complete measures of place attachment (Lewicka, 2008) and social support (Sarason et al., 1987).

### Loneliness

Loneliness was measured using the 20-item UCLA Loneliness Scale (Russell et al., 1980). Three questions of the twenty have been identified as an adequate short-form measure for loneliness (Hughes et al., 2004). Due to missing data for the full 20-item measure, Multiple Imputation (*m* = 10) was used to derive scores for participants that had completed more than 60% of the 20-item measure. Imputed responses to the three questions of the short-form measure were utilized as the measure for loneliness in this study. Participants with randomly missing data for any of the other variables were removed from analysis only for the individual tests where their response was missing.

### Year-by-Year Interactions

A set of questions asked for the amount of time, in hours, that participants spent in various kinds of interactions on an average day the previous week (Fall, 2020), separating out interactions taking place via instant messaging, voice calls, video calls, in-person with roommates or housemates (called roommates, in this study), and in-person with individuals that do not live with them. Students were the asked to recall the time spent in each type of interaction during a typical week. This set the year prior (Fall, 2019) before the emergence of the pandemic. In addition, the survey asked participants to identify three physical places in which most of their in-person interactions occurred both in the previous week and a typical week a year prior.

### Social Interaction Meaningfulness

The quality of recent social interactions was assessed by examining self-reported meaningfulness, using five dimensions (self-disclosure, other-disclosure, intimacy, meaning, satisfaction) of the Rochester Interaction Record (RIR) which have been used previously as a measure for how meaningful an interaction was (Wheeler et al., 1983). This scale was altered slightly to account for modern meanings of the measure’s original language (e.g., *meaningfulness* used instead of *intimacy*). Students rated the meaningfulness of (1) “a typical interaction with your friends or peers last week” and (2) their most recent interaction.

### Details of Most Recent Interaction

For their most recent interaction, participants were asked to include additional details, including whether it occurred in a private or public space (i.e., “privacy”), if it was in-person or virtual (i.e., “mode”), their relationship to each person in the interaction (i.e., “relationship”), and if the interaction had been scheduled in advance or was spontaneous (i.e., “planned”). Additionally, they were asked to report every type of activity that was part of the interaction (i.e., “activity”; e.g., eating, working).

The study and its survey tool were approved by the Institutional Review Board of the participating university, with participants providing informed written consent prior to participating in the study.

## Results

The survey was completed by 132 college students with an average age of 21 years (*SD* = 3.88 years), the majority of whom were female (80%) and lived at the time in the same state as the university (84%) (see Table 1 for participant demographics). Of those students who had returned to the city, most lived off-campus in a range of housing types.

**TABLE 1:** Demographic variables.

Category	#	%
**Gender**		
Female	106	80.3
Male	24	18.2
Other	2	1.5
**Age**		
18	24	18.0
19	27	20.5
20	28	21.2
21	23	17.4
22	4	3.0
23	4	3.0
24	3	2.3
25	3	2.3
26	0	0.0
27	2	1.5
28	4	3.0
29	1	0.8
30	2	1.5
31	1	0.8
32	1	0.8
33	2	1.5
34	2	1.5
35	1	0.8
**Years at this University**		
1	35	26.5
2	39	29.6
3	31	23.5
4	23	17.4
5	2	1.5
>5	2	1.5
**Current place of living**		
**Number of people live with**		
0	27	20.5
1	37	28.0
2	21	15.9
3	14	10.6
4	16	12.1
5	8	6.1
6–10	5	3.8
>10	3	2.3
Unknown	1	0.8
**Relationship to people living with**		
Alone	27	20.5
With significant other	7	5.3
With family	28	21.2
With roommates	22	16.7
With housemates	56	42.4
**Location: Country/State**		
In state	111	84.1
In United States not in state	14	10.6
Not United States	7	5.3
**Location: For participants in state**		
In the city	101	91.0
≤60 miles from city	1	1.0
>60 miles from city	9	8.9
**Location: Relative to campus**		
On campus	37	36.6
Off campus	64	63.4
**Residence type**		
Dorm	35	26.5
Single	28	21.2
Duplex	8	6.1
Multi ≤4 floors	36	27.3
Multi >5 floors	20	15.2
Other	4	3.0
NA	1	0.8
**Years lived there**		
<1	79	59.9
1 to <2	20	15.2
2 to <3	6	4.6
3 to <4	2	1.5
4 to <5	0	0.0
5 to <10	2	1.5
10 to <15	4	3.0
15+	14	10.6
Unknown	5	3.8
**Do you consider this “home?”**		
I don’t know	1	0.8
Definitely	42	31.8
Somewhat	70	53.0
Not at all	19	14
**Is this your permanent residence?**		
Yes	35	26.5
No	96	72.7
Unknown	1	0.8
**Total**	132	

When asked to list the total number of individuals that they could turn to provide social support participants reported an average of 6 people. Participants indicated a moderately high level of satisfaction with the amount of social support in their lives (Table 2). Feelings of loneliness among participating students were relatively high, with a mean score of 7.79 on a scale of three (low loneliness) to twelve (high loneliness). A Pearson’s product moment correlation found a significant negative correlation between age and loneliness [*r* = −0.29, *t*(97) = −3.01, *p* = 0.003], but no significant correlation between years of education at the university and loneliness [*r* = −0.11, *t*(97) = −1.14, *p* = 0.26]. The correlation between loneliness and meaningfulness of a typical interaction last week approached significance [*r* = −0.26, *t*(46) = −1.81, *p* = 0.08], and a significant correlation emerged when examining loneliness and meaningfulness of the most recent interaction [*r* = −0.35, *t*(52) = −2.67, *p* = 0.01].

**TABLE 2:** Social support and loneliness.

	Range available	*M*	*SD*
Average # of people providing social support	Open: no cap	5.66	*4.24*
Satisfaction	1 (high) to 6 (low)	2.22	*1.03*
Feelings of loneliness	3 (low) to 12 (high)	7.79	*2.45*

*M, Mean; SD, Standard Deviation.*

**FIGURE 1:**
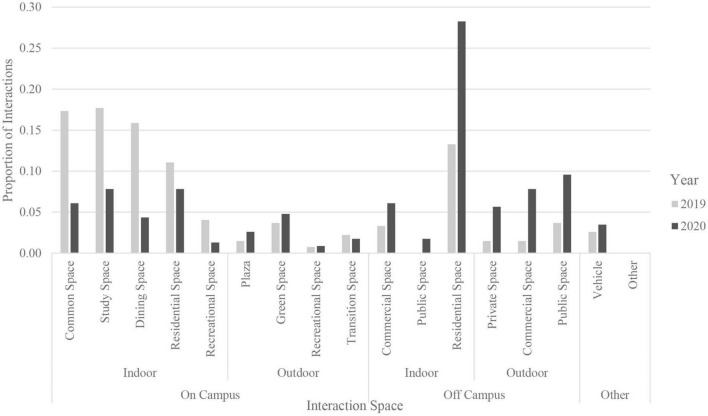
Proportion of in-person interactions in three most common spaces, excluding first year students.

*T*-tests found that, in 2020, participants spent significantly more time [*x* = 1.87 h, *t*(128) = 7.22, *p* < 0.001] interacting in-person in than they did via video calls, when including both interactions with those that they lived with and individuals outside of the home (see Tables 3, 4). Hours spent in interactions with housemates or roommates on an average day did not differ significantly between 2020 and 2019 [*x* = −0.05, *t*(129) = −0.28, *p* = 0.78]. However, students spent significantly less time interacting in-person with people other than house/roommates in 2020 [*x* = 1.25, *t*(127) = 6.97, *p* < 0.001] then the same time the previous year. Examinations of year-by-year interactions mediated by technology found no significant differences in the hours spent instant messaging per day [*x* = −0.001, *t*(129) = −0.01, *p* = 0.99], while participants did spend significantly more time interacting in 2020 over voice [*x* = 0.31, *t*(128) = 3.56, *p* < 0.001] and video call [*x* = 0.82, *t*(128) = 0.61, *p* < 0.001] than they did the year prior. Examination of students’ three most common spaces for in-person interactions in 2020 and 2019 found a large reduction in the reported use of on-campus indoor spaces, a moderate increase in the use of the majority of outdoor spaces, and a large uptick in the use of residential spaces during the pandemic, with similar trends for all participants and when first-year students are excluded from the analysis (Figure 1).

**TABLE 3:** *T*-tests of time spent interacting between 2019 vs. 2020.

Interaction activity	Estimate	*t*	df	*p*-value	95% CIs
In-person with housemates	−0.05	−0.28	129	0.78	(−0.41, 0.30)
In-person with others	1.25	6.97	127	<0.001**	(0.90, 1.61)
Via voice call	−0.31	−3.56	128	<0.001**	(−0.48, −0.14)
Via video call	−0.82	−0.61	128	<0.001**	(−1.08, −0.55)
Via messaging	−0.001	−0.01	129	0.99	(−0.26, 0.26)

**p < 0.01.

**TABLE 4:** Average hours per day spent interacting in 2019 vs. 2020.

	2019		2020	
Interaction activity	*M*	*SD*	*M*	*SD*
In-person total	4.71	*2.84*	3.48	*2.77*
In-person with housemates	1.85	*1.94*	1.90	*1.99*
In-person with others	2.83	*1.77*	1.59	*1.71*
Via voice call	0.57	*0.88*	0.86	*1.17*
Via video call	0.75	*0.92*	1.57	*1.55*
Via messaging	1.83	*1.51*	1.83	*1.59*

*M, Mean; SD, Standard Deviation.*

### Most Recent Interaction

**FIGURE 2:**
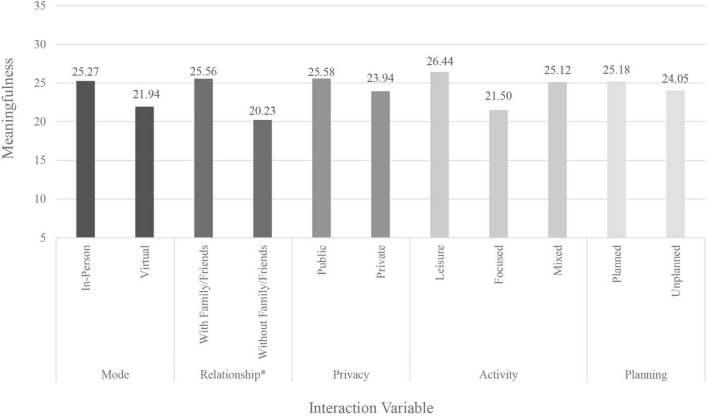
Mean score for loneliness by interaction variable. *Represents *p* < 0.05 when variable tested independently.

A two-sample *t*-test found that participants reported significantly higher meaningfulness [*t*(66) = −2.42, *p* = 0.018] for interactions that were with family members and/or friends (*M* = 25.56) compared to those not including family or friends (*M* = 20.23) (see Figure 2 and Table 5). A 2 × 2 analysis of variance (ANOVA) found a significant main effect for mode of the interaction on meaningfulness [*F*(1,65) = 10.21, *p* = 0.002], and a significant main effect of whether the interaction was planned [*F*(1,65) = 4.70, *p* = 0.03; Table 6]. Examination of the significant mode by planning interaction effect [*F*(1,65) = 6.49, *p* = 0.01] found that virtual interactions that were planned had the lowest meaningfulness scores (*M* = 20.0) and scores for planned interactions in-person had the highest meaning (*M* = 28.9), with scores for unplanned interactions falling between the two (*M*_in–person_ = 25.5, *M*_virtual_ = 23.8) (see Table 7).

**TABLE 5:** *T*-tests of meaningfulness by relationship to person/people and privacy level.

			*t*	df	*p*-value	95% CIs
	*With family/friends*	*Non-family/friends*				
Relationship	25.56	20.23	−2.42	66	0.02*	(−9.73, −0.94)
	*Public*	*Private*				
Privacy	25.58	23.94	0.82	66	0.42	(−2.38, 5.66)

*p < 0.05.

**TABLE 6:** Two-way ANOVA for meaningfulness.

	*SS*	*df*	*F*	*p*-value
Planning	229.3	1	4.7	0.03*
Mode	498.9	1	10.21	0.002**
Mode × Planning	317.2	1	6.49	0.01*
*Residuals*	3174.9	65		
Activity type	180.2	2	1.7	0.19
*Residuals*	3436.3	66		
Building type	575.9	11	0.95	0.50
*Residuals*	3131.2	57		

*p < 0.05, **p < 0.01.

**TABLE 7:** Simple main effects on meaningfulness for planning by mode.

Interaction	*M*	SE	df	*t*	*p*-value	95% CIs
*Virtual*						
Planned	20.0	2.11	65	9.49	<0.001**	(15.8, 24.2)
Unplanned	25.5	2.85	65	8.94	<0.001**	(19.8, 31.2)
*In-person*						
Planned	28.9	1.8	65	16.00	<0.001**	(25.3, 32.5)
Unplanned	23.8	1.15	65	20.72	<0.001**	(21.5, 26.1)

**p < 0.01.

**FIGURE 3:**
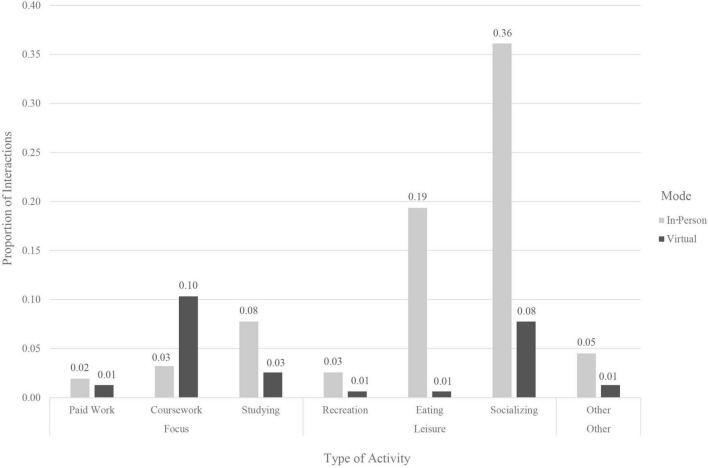
Proportion of type of activity occurring during interaction.

**FIGURE 4:**
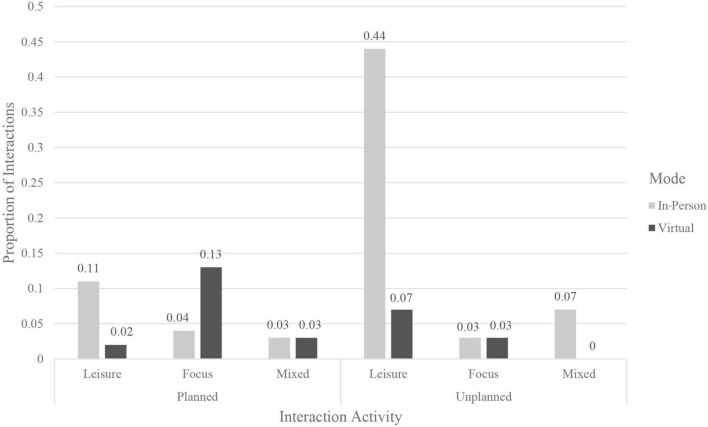
Proportion of types of interactions, by mode and planning.

A one-way ANOVA found no significant differences [*F*(2,66) = 1.70, *p* = 0.19] in reported meaningfulness based on the nature of the activity occurring during the interaction (i.e., leisurely, focused, or a mix of both) (see Table 6 and Figures 3, 4). A two-sample *t*-test found no significant differences [*t*(38.15) = 0.87, *p* = 0.42] for meaningfulness between interactions occurring in private (*M* = 23.94) or public settings (*M* = 25.58) (Table 5).

**FIGURE 5:**
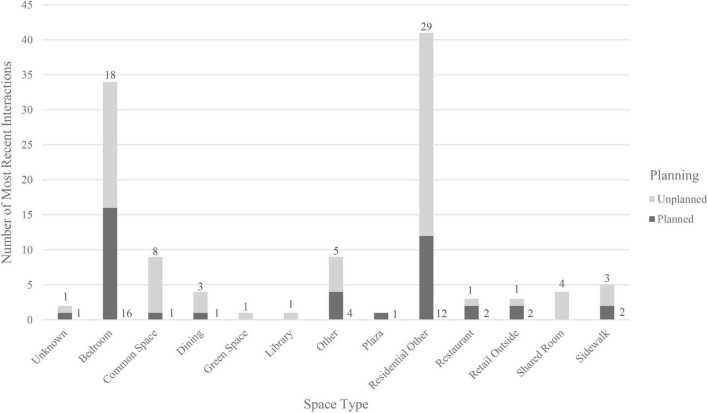
Number of total interactions, by space type and planning.

An ANOVA examining the types of spaces in which both virtual and in-person interactions occurred found no significant differences in meaningfulness of the interaction [*F*(11,57) = 0.95, *p* = 0.50; Table 6]. However, there were large differences between the total number of interactions that occurred within each space type (e.g., *n*_bedroom_ = 34, *n*_library_ = 1; see Figure 5).

## Discussion

The COVID-19 pandemic altered the lives and routines of individuals all over the globe. This study demonstrates that the pandemic also had profound impacts on daily social interaction patterns among college students, mirroring patterns of reduced social contact found in a recent meta-analysis (Liu et al., 2021), but contrasting a finding of no differences in the amount of time spent interacting after the implementation of restrictions early in the COVID-19 pandemic (Fried et al., 2022). Participating students spent significantly less time in face-to-face interactions with individuals other than roommates, and more time spent on voice and video calls. These changes are not surprising given the physical distancing measures and limits to amount of social contact that were in place, and often mandated, in order to limit the spread of COVID-19 (Sen-Crowe et al., 2020). Time spent the year prior on in-person interactions such as class, work, and socializing with friends was supplanted, although not entirely, with time spent interacting virtually. Even with the increase in time spent in video calls, the overall time that students spent in 2020 interacting with others in-person was still greater than the time spent interacting over video calls, despite the significant drop in in-person interactions with non-roommates. However, these year-to-year reductions in in-person interactions account for over an hour a day of lost time spent interacting in-person. Considering the substantial shift from in-person to video/voice calls, and the care with which individuals had to select with whom and where they would interact in-person during the pandemic, it is vital to examine the aspects of interactions that could be important for facilitating positive well-being and opportunities for self-care during continuing restrictions and beyond. College students face significant barriers to high-quality social interactions even outside the pandemic, as they navigate stressful and often busy schedules and must be selective when deciding how, and with whom, to spend their time. Therefore, determining which components of interactions are linked to higher meaningfulness can help inform individuals when making interaction decisions, as well as the provision of spaces which support meaningful interactions. Some physical spaces that were traditionally reserved for specific kinds of interactions, such as the use of classrooms for instruction, were completely abandoned during the height of the pandemic. Other spaces, especially those often designated for private life and restoration, were invaded by different types of virtual interactions (e.g., bedrooms and living rooms became places to attend virtual classes and meetings). We can see this through in shifts toward interactions inside the home in 2020 compared to 2019. When considering spaces related to self-care practices (e.g., nail salons, recreational spaces) and those in which social interactions and self-care activities often take place (e.g., restaurants), many of these settings were either highly restricted or unavailable altogether (Storr et al., 2021). In this way, the lack of availability of activities and spaces which can be used for self-care practices necessitated a shift toward other types of activities or different spaces in which regular self-care activities could take place.

Meaningful interactions, those that can contribute to positive self-care, stronger social ties, and lower loneliness, were linked to some specific interaction features in this study. The differences in meaningfulness of almost 30% between planned virtual and planned in-person interactions relate to the fact that most of the planned virtual interactions were for work- or study-related activities and the majority of planned in-person interactions constituted social engagements and more leisurely activities. The results here indicate that planned in-person interactions could be vital for maintaining existing self-care patterns, engaging in self-care activities (e.g., taking walks), and receiving the kinds of support and attention that are not available to the same extent when interacting virtually. While the low percentage of focused interactions occurring in-person is likely linked to pandemic-related restrictions, such a large proportion of leisure activities taking place in-person and not virtually is likely not just due individuals avoiding Zoom fatigue (Fauville et al., 2021) but hints at the importance of interaction characteristics and experiences available only when interactions are in-person. Though work examining different modes of face-to-face interactions is limited, some scholars suggest that physical co-presence plays a role in the perceptions of the interaction (e.g., Schroeder, 2002) and work in physical propinquity has found ties to social interaction opportunities and the formation of friendships (Nahemow and Lawton, 1975). Although previous research has found that online interactions can also provide social support in ways that promote better well-being (Canale et al., 2021), current technological advancements allow only limited opportunities for engaging in fulfilling self-care activities in the virtual sphere, as compared to those afforded by a physical space. One of the measures adopted by many during the pandemic was the creation of a Covid “social bubble,” or group of individuals that all agreed to interact with each other, while staying distant from those outside their group (Leng et al., 2020). Modern technology provided opportunities to maintain connection with individuals outside our bubble through virtual interactions, voice calls, and messaging. If in-person planned interactions are most meaningful, with interactions with family and friends being linked to the best quality outcome, then in times of limited in-person contact, this study suggests students should prioritize those with whom they have the most intimate relationships. Other research has found evidence that individuals during the pandemic adopted this strategy, choosing to spend time with those with whom they have stronger social ties (Storr et al., 2021). Similarly, the choice to move back home, for many students an involuntary decision, may have led to better outcomes than if students had stayed in their pandemic bubbles on campus while living with randomly assigned roommates, because of the increased strength of the ties and social support available. However, it is possible that physical disconnection from friends who help form social support networks, especially for those experiencing time zone differences, led to heightened feelings of loneliness and fewer opportunities for social interaction and self-care activities. Here, there is potential for future work to explore the links between types of people with whom we live and the outcomes of meaningfulness of interactions, loneliness, and social support.

Unexpectedly, no link was found between differences in meaningfulness and the type of interaction activity, that is, various leisure versus focused activities. Some types of activity were fairly restricted compared to pre-Covid opportunities, especially for activities of leisure which are often also tied to self-care practices (e.g., visiting hair and nail salons). This lack of difference may reflect that leisure activities during the pandemic were likely to be closer in character to the activities of studying and working, especially for activities occurring virtually, and so presented similar patterns. Pandemic restrictions during Fall 2020 meant there was a decreased ability to access, and possibly heightened anxiety around the use of, public spaces for leisure. This may also help explain why interactions occurring in private versus public spaces did not vary significantly in meaningfulness. Even when participants were able to interact with each other in public, many regulations and space features likely detracted from quality of the interaction (e.g., socially distanced furniture, mask regulations). Future work could more deeply examine the ways in which public and private settings can each be conducive to high-quality interactions. The physical spaces themselves were not linked to meaningfulness in this study, likely due to the limited range of spaces in which participants recorded having interactions that day, a consequence of pandemic-related restrictions. Though meaningfulness was not significantly different based on types of interaction spaces used in the most recent interaction, work on the importance of Third Place and neighborhood characteristics suggests that the findings here might not be capturing a large enough diversity of spaces necessary to detect effects of physical setting characteristics on meaningfulness with sufficient power. While other characteristics of the interaction were tested using less than 5 levels for any individual variable, a larger number of space types were available for participants to choose from, making it more difficult to detect a statistically significant result.

The moderately high feelings of loneliness found among college students in this study mirror not only other pandemic findings, but also the larger trend of increasing loneliness among this population and the associated age group. Correlations found between meaningfulness of the most recent interaction and loneliness, with close to significant links between the meaningfulness of the previous week’s interactions and levels of loneliness support the findings of prior research (Wheeler et al., 1983) and point to the importance of understanding the implications of daily interaction opportunities and decisions.

This study had several limitations, primarily related to the participant sample. Eligible college students self-selected to be in this study, and an overwhelming majority of participants were female. Since recruitment materials mentioned social interactions explicitly, it is possible that those feeling vulnerable about discussing their social interactions (e.g., particularly lonely individuals) actively chose to not participate in the study, leading to discrepancies between the types of interactions and outcomes captured within the study and those that typify the greater college student population. Therefore, the results of this work should be replicated and furthered with a larger, more diverse, and representative sample. Lastly, despite alignment with findings from previous research, the design of the study precludes the ability to make claims regarding causation and is limited in its’ conclusions regarding the direction of the relationship between social interaction meaningfulness and loneliness.

## Conclusion

Meaningful social interaction is both a type of self-care practice and an avenue for promoting positive self-care behaviors. Understanding the characteristics of the most meaningful interactions can help guide strategies for performing self-care activities and inform decisions regarding social interactions in the future. Self-care activities, including social interactions, were often limited or restricted during the COVID-19 pandemic. College students, a group of individuals already identified as experiencing high level of loneliness prior to the pandemic, experienced enormous changes in their daily social interactions. Whether during the pandemic or beyond, college students may be able to buffer against loneliness by strategically prioritizing the people, activities and conditions which are more likely to produce meaningful social interactions within their busy and stressful schedules. This may be particularly important in the short term when options for in-person interactions remain limited for some and Zoom fatigue is still an issue. While meaningful social interactions are in and of themselves a self-care strategy that can help improve mental health outcomes, mitigate loneliness, and bolster social support for college students, they also are avenues for promoting self-care activities.

University and government bodies can also help to mitigate loneliness by stressing the importance of focusing on interactions that promote self-care and health, particularly with those that provide social support and encourage self-care strategies. Considering the findings of this work and the recent emphasis on combating loneliness made by policy makers, researchers, and journalists, research in this area should continue to examine the factors with can promote more meaningful interactions in more depth. Further work in this area should uncover and examine if meaningfulness of an interaction with a close contact would be affected by the mode of the interaction, such as in-person versus virtual, including working to understand the mechanisms through which different modes of interaction impact outcomes. Future work should also examine in more depth the influence of the environmental setting of daily interactions on meaningfulness or loneliness outcomes, including consideration of which environmental or spatial characteristics can contribute to or detract from the quality of social interactions. This increased examination of the conditions that can facilitate more meaningful social interactions will allow researchers, institutions, and individuals to create strategies and make decisions in order to help promote effective self-care practices and mitigate loneliness among college students and other young adults.

## Data Availability Statement

The raw data supporting the conclusions of this article will be made available by the authors, without undue reservation.

## Ethics Statement

The studies involving human participants were reviewed and approved by the Cornell University Institutional Review Board. The participants provided their written informed consent to participate in this study.

## Author Contributions

RB involved in the preparation of the questionnaire, data collection and analysis, and manuscript writing. JL involved in advising the research direction, guiding the questionnaire formulation, and article revision. Both authors contributed to the article and approved the submitted version.

## Conflict of Interest

The authors declare that the research was conducted in the absence of any commercial or financial relationships that could be construed as a potential conflict of interest.

## Publisher’s Note

All claims expressed in this article are solely those of the authors and do not necessarily represent those of their affiliated organizations, or those of the publisher, the editors and the reviewers. Any product that may be evaluated in this article, or claim that may be made by its manufacturer, is not guaranteed or endorsed by the publisher.
